# Supplementary material for: Investigation of α-Glucosidase Inhibitory Metabolites from Tetracera scandens Leaves by GC–MS Metabolite Profiling and Docking Studies
Source: Biomolecules. 2020 Feb 12;10(2):287. doi: 10.3390/biom10020287 (PMC7072363; doi:10.3390/biom10020287)
Supplement: Supplementary file 1 [file biomolecules-10-00287-s001.zip › Proofed Supplementary/biomolecules-622424 -proofed--Supplementary data (File S2).docx]

# PREDICTED INTERACTIONS OF BEST DOCKED CONFORMATION – ENZYME COMPLEXES


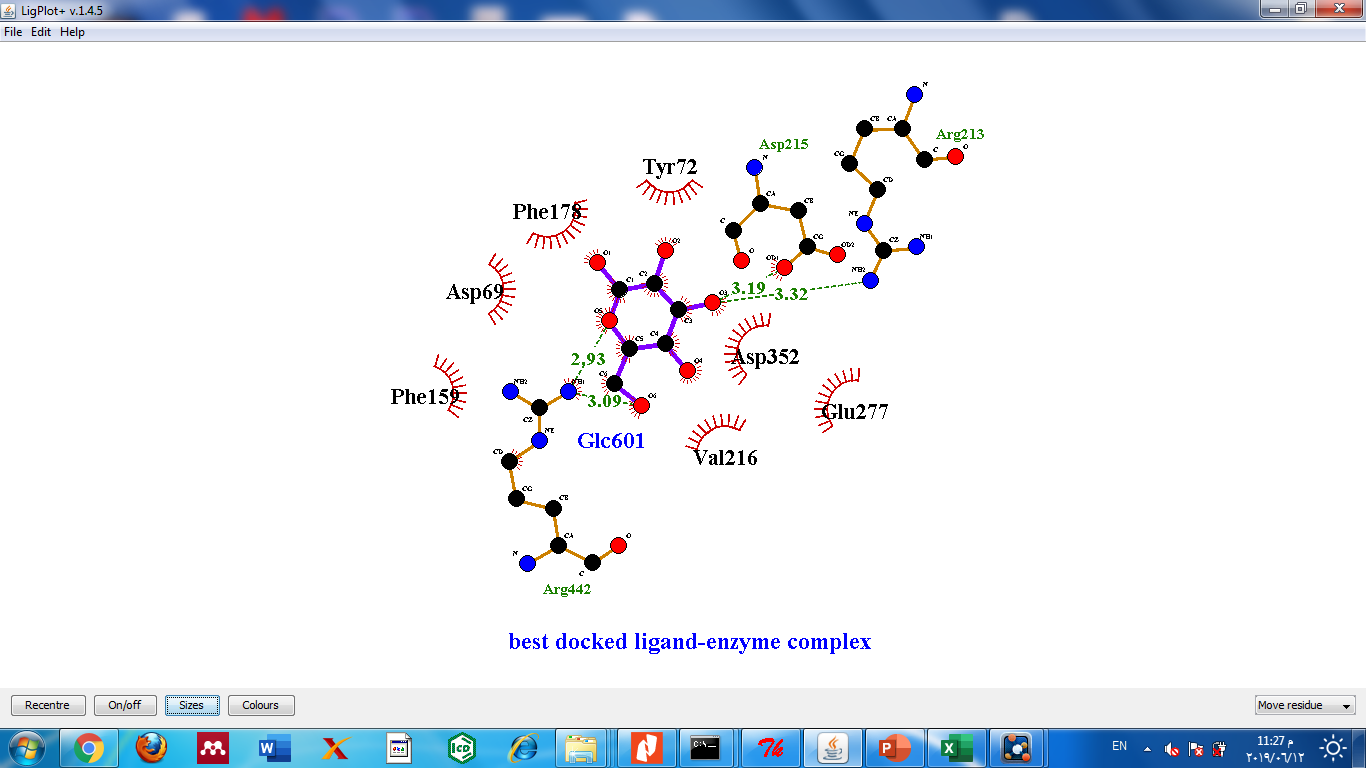


**Control ligand**


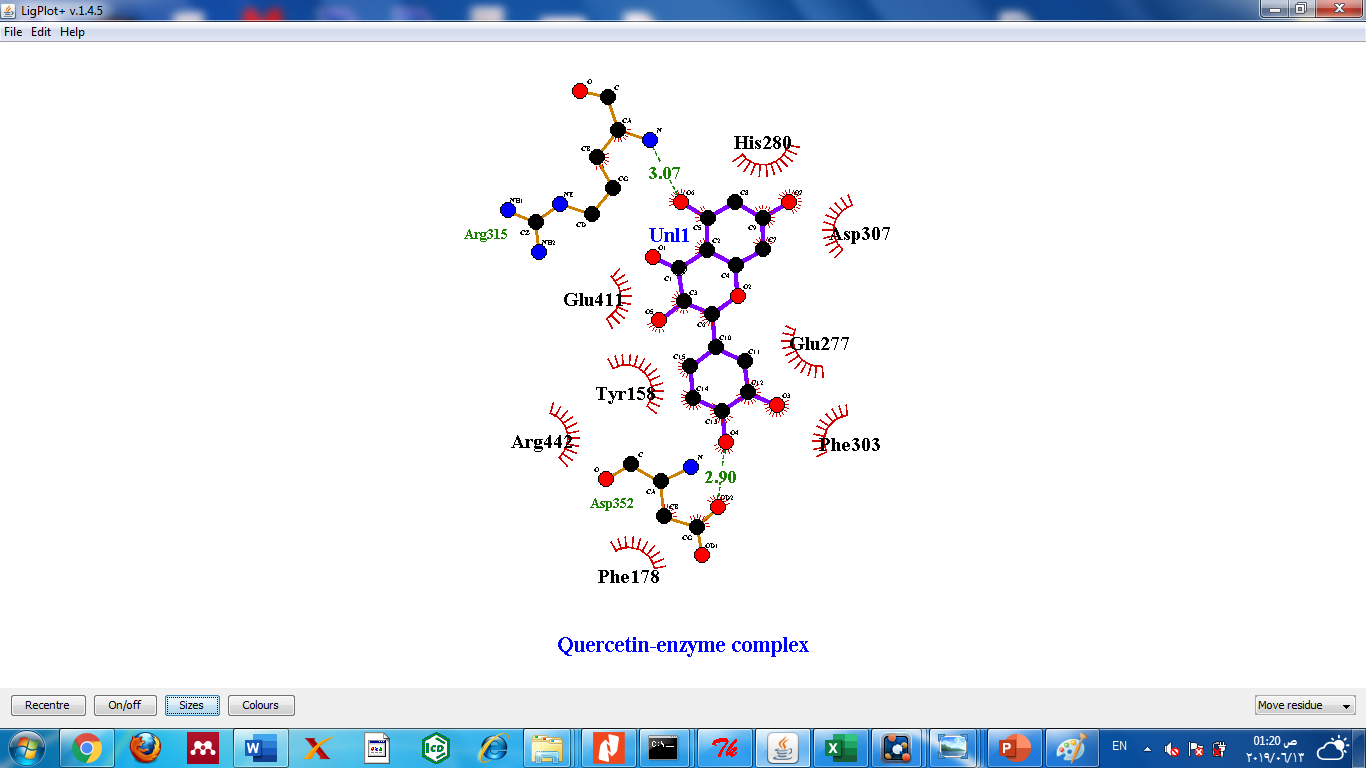


**Quercetin**


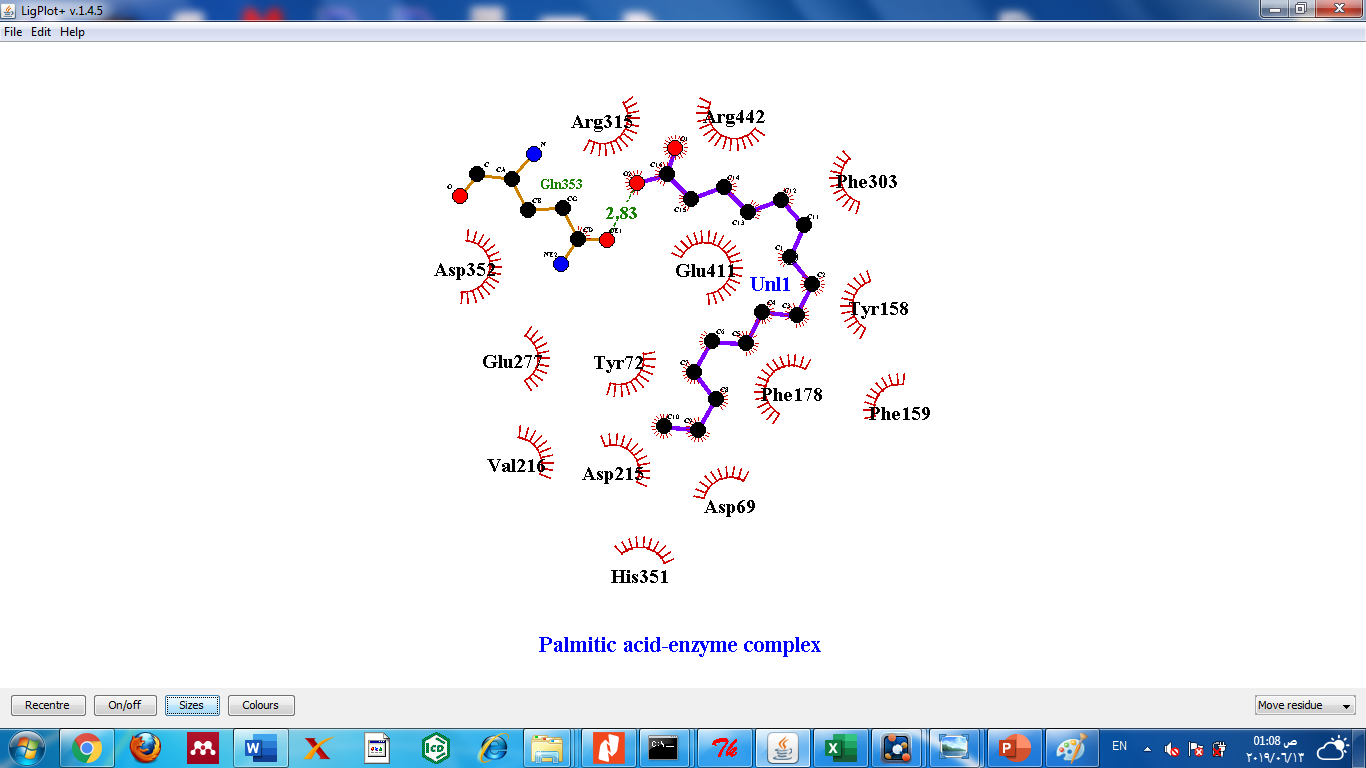


**Palmitic acid**


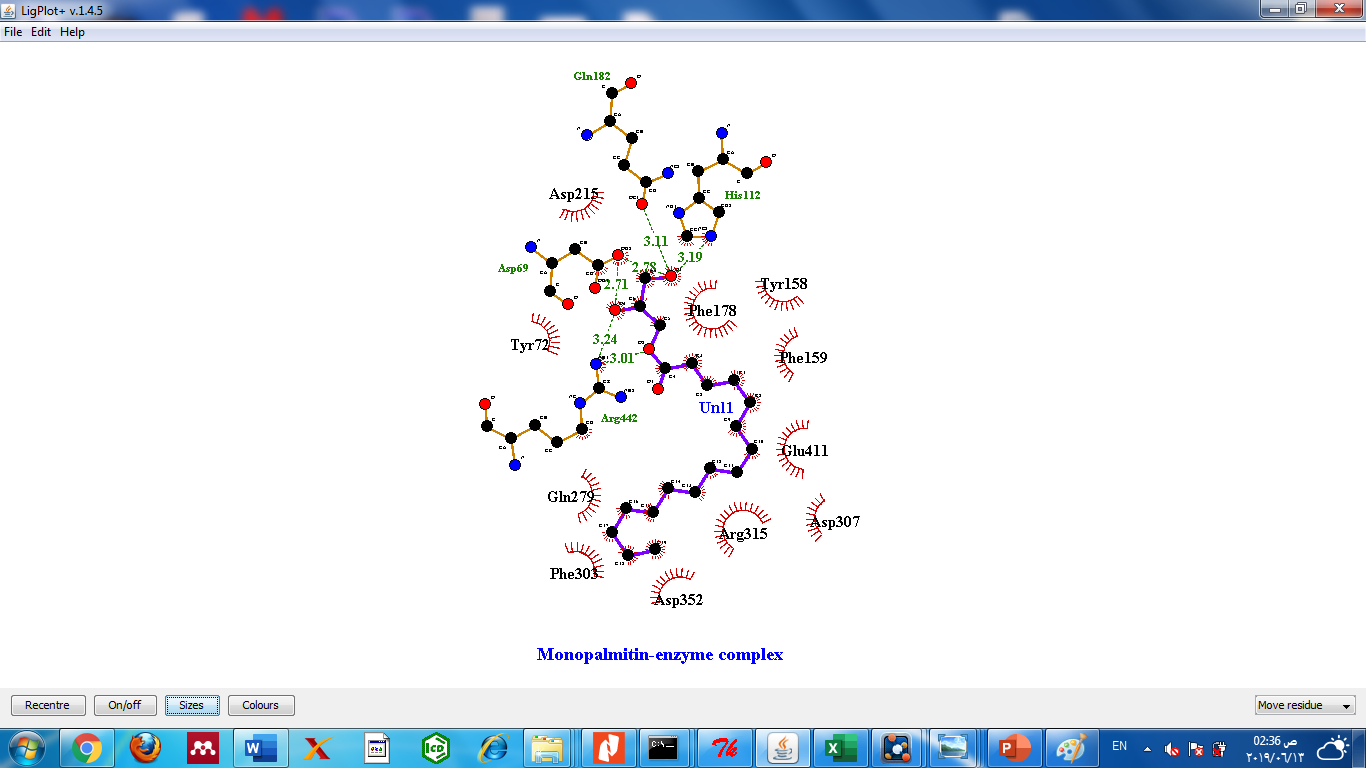


**1-Monopalmitin**


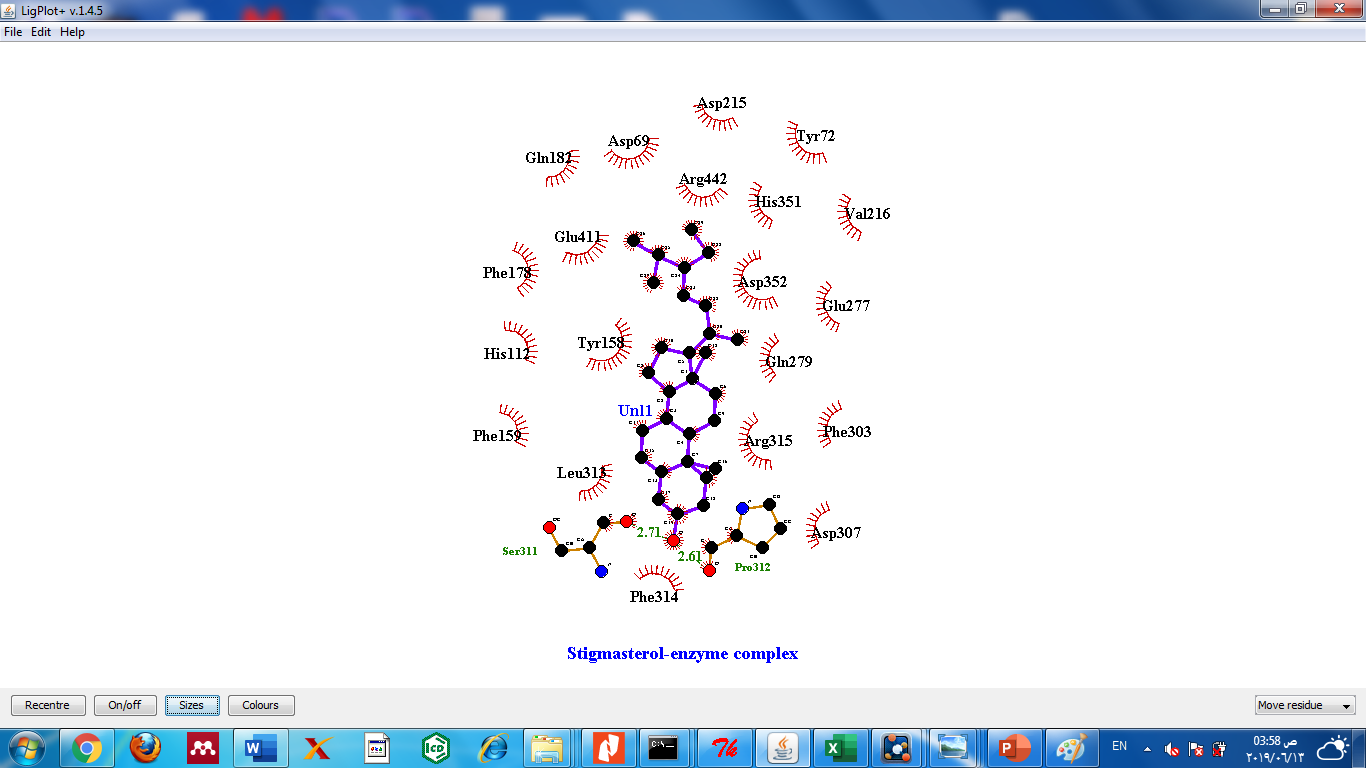


**Stigmasterol**


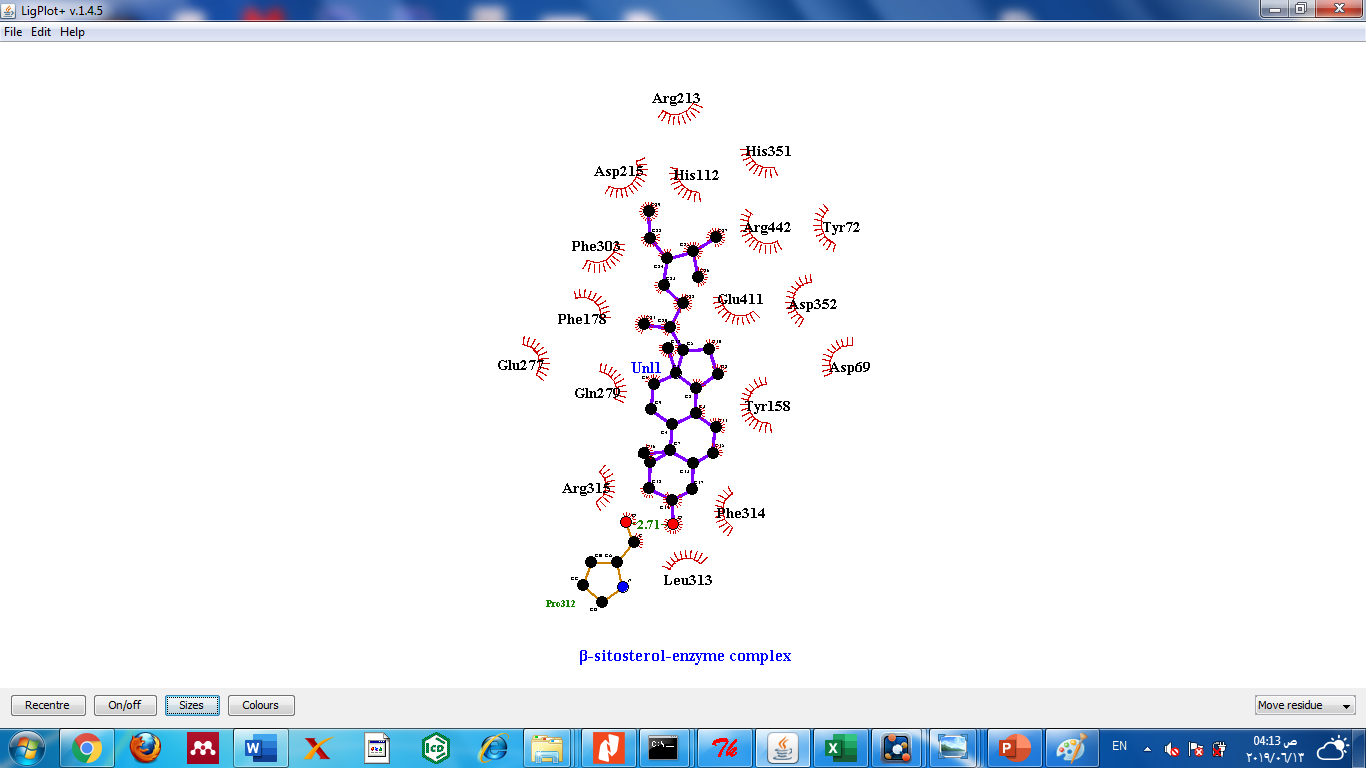


**β-Sitosterol**


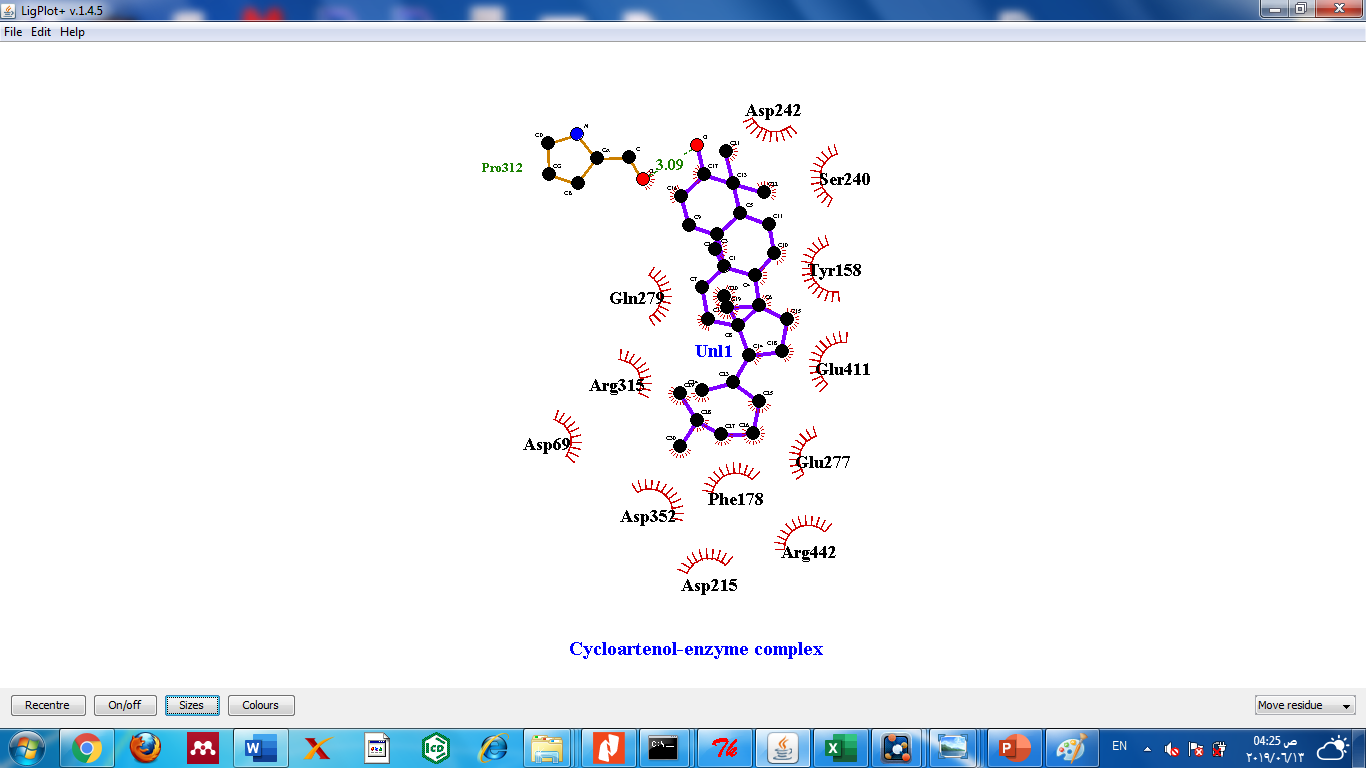


**Cycloartenol**


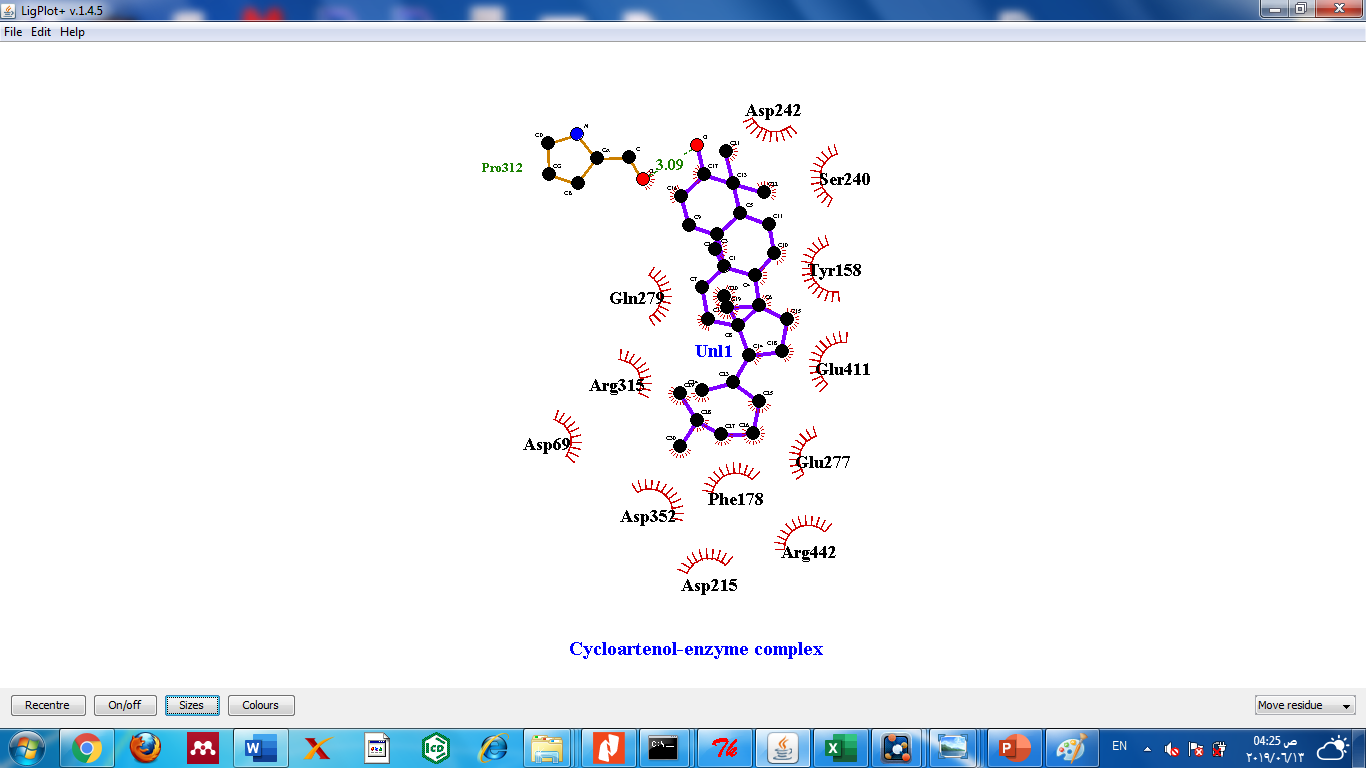


**24-Methylenecycloartenol acetate**


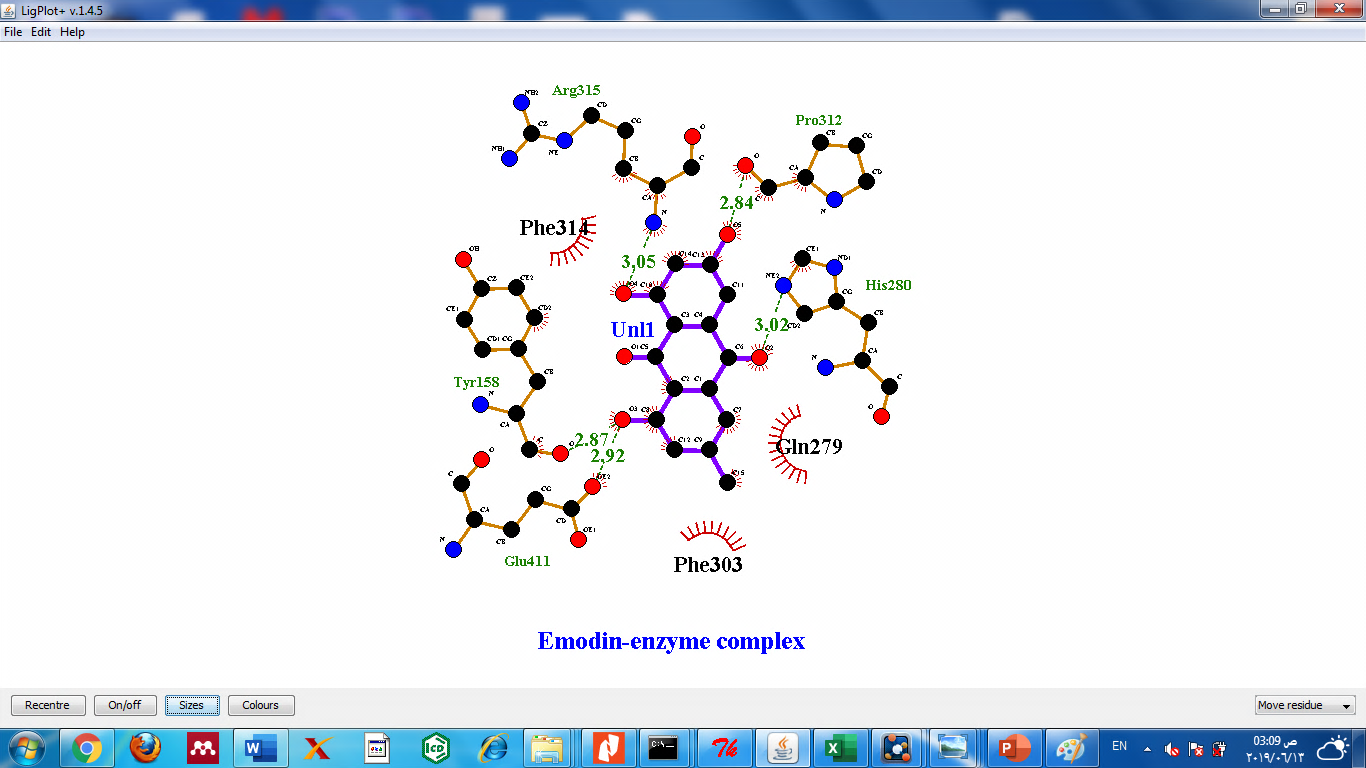


**Emodin**


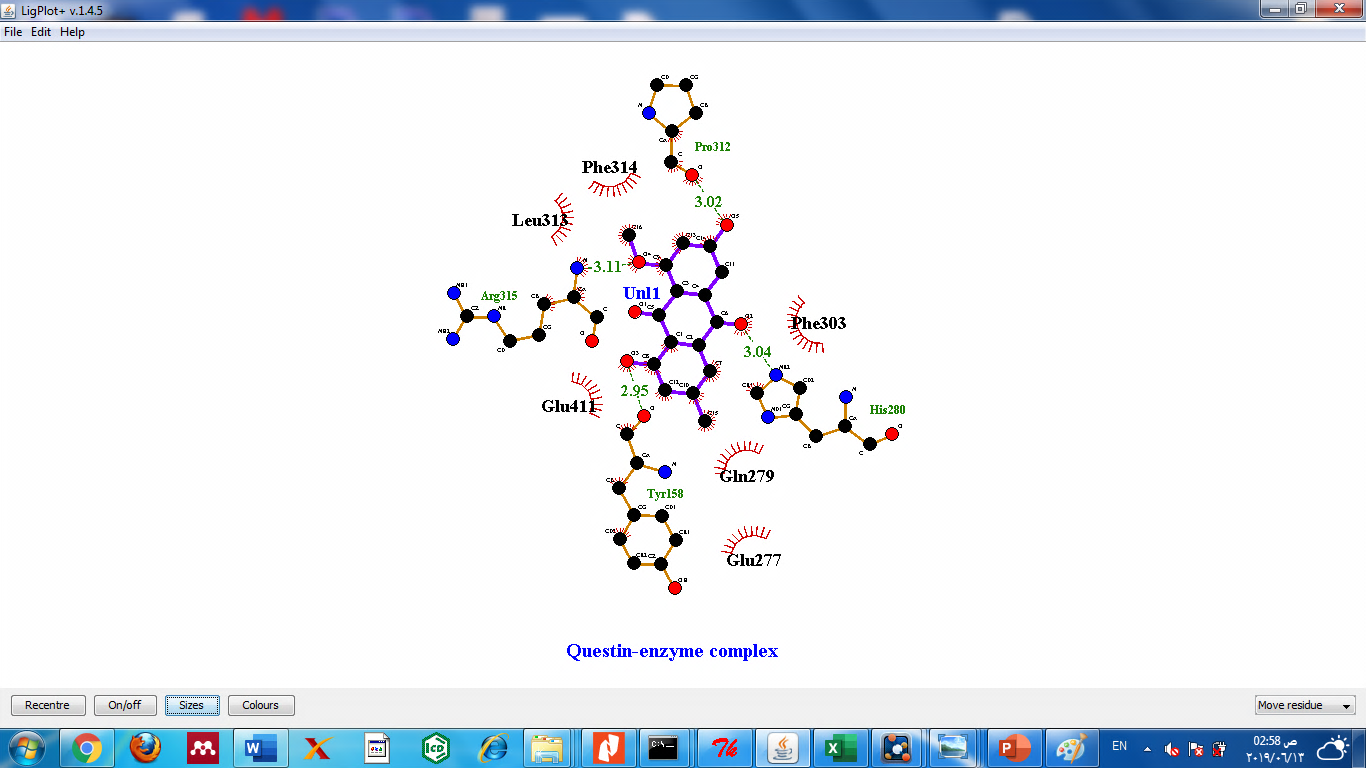


**Questin**


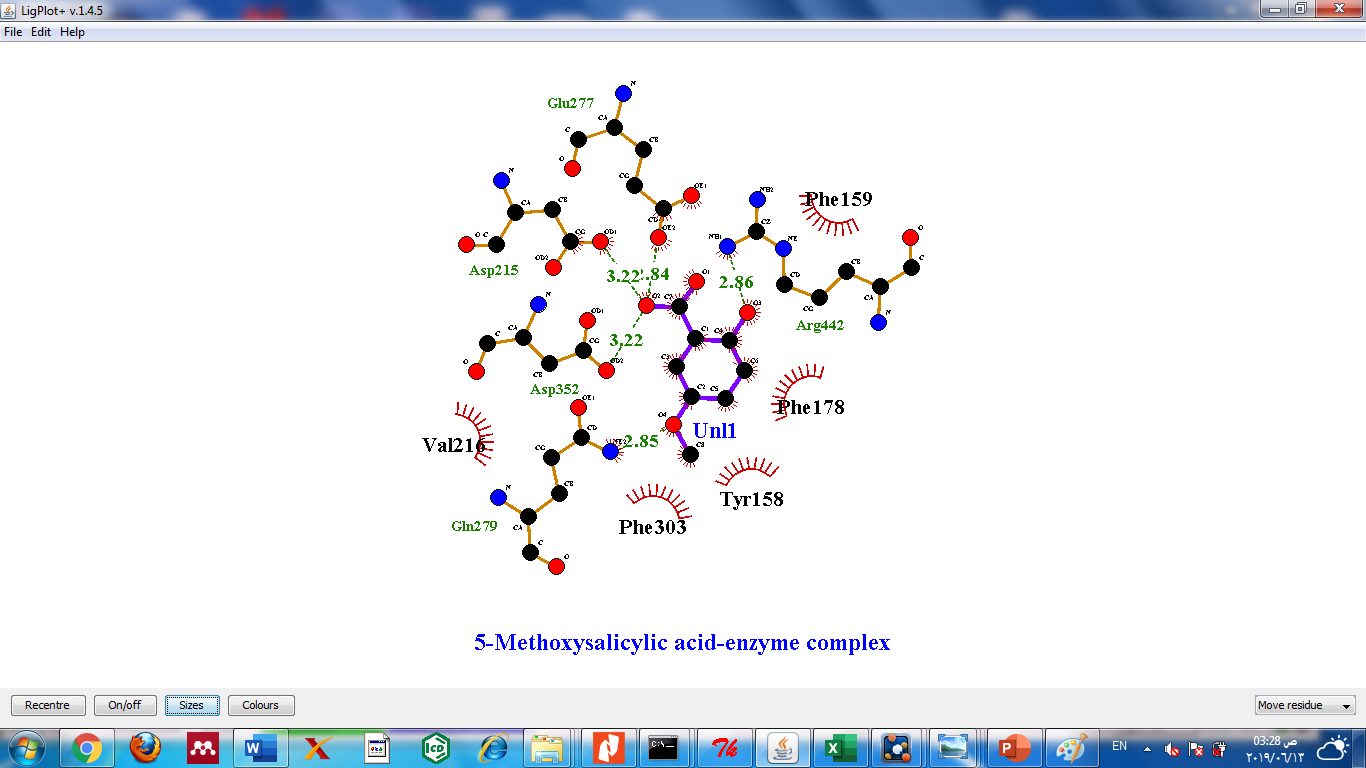


**5-Methoxysalicylic acid**


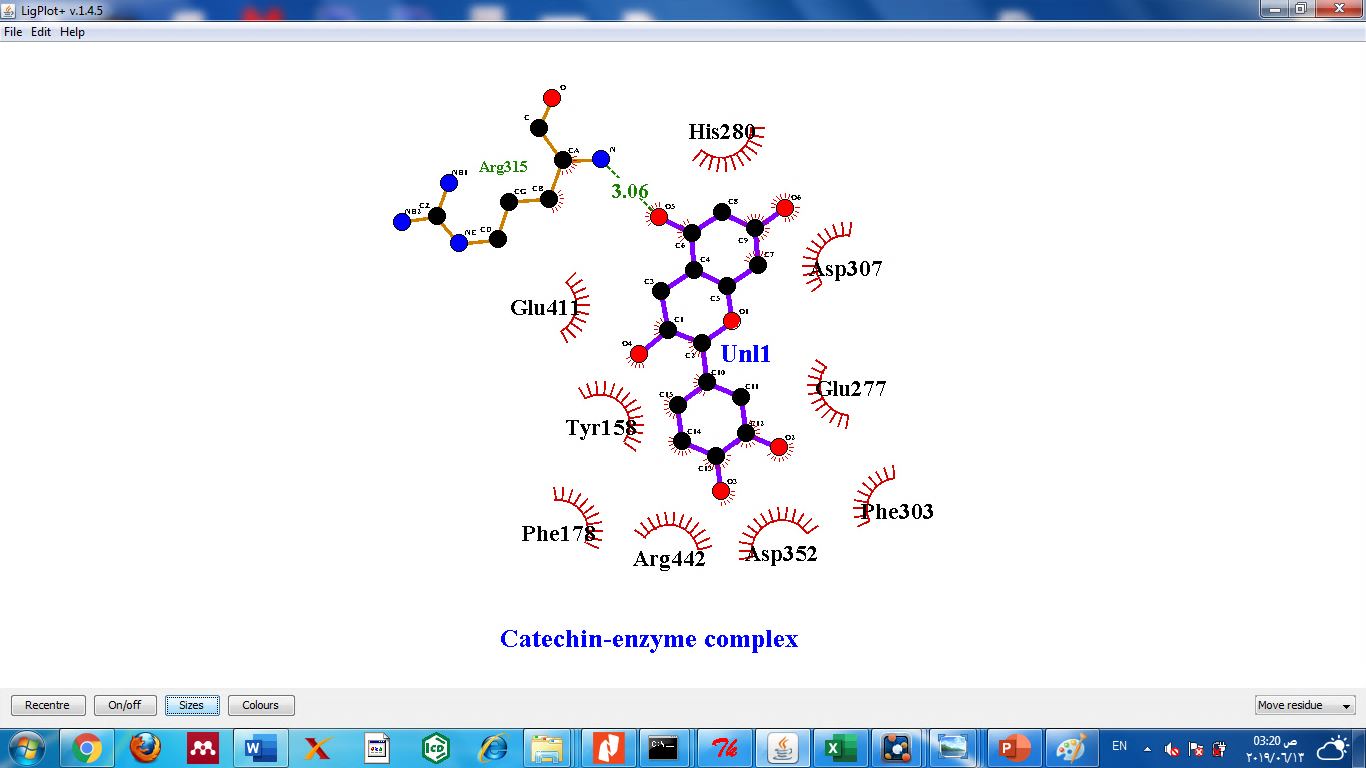


**Catechin**


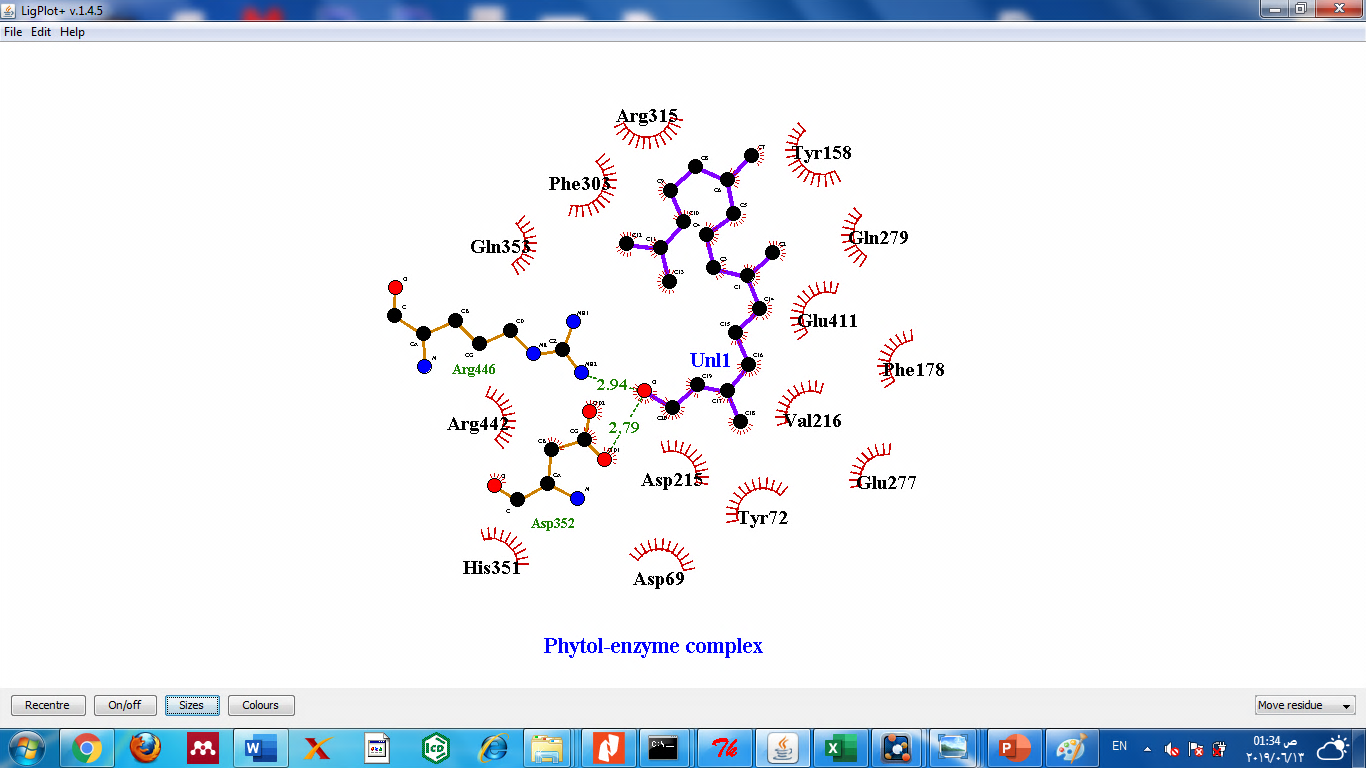


**Phytol**


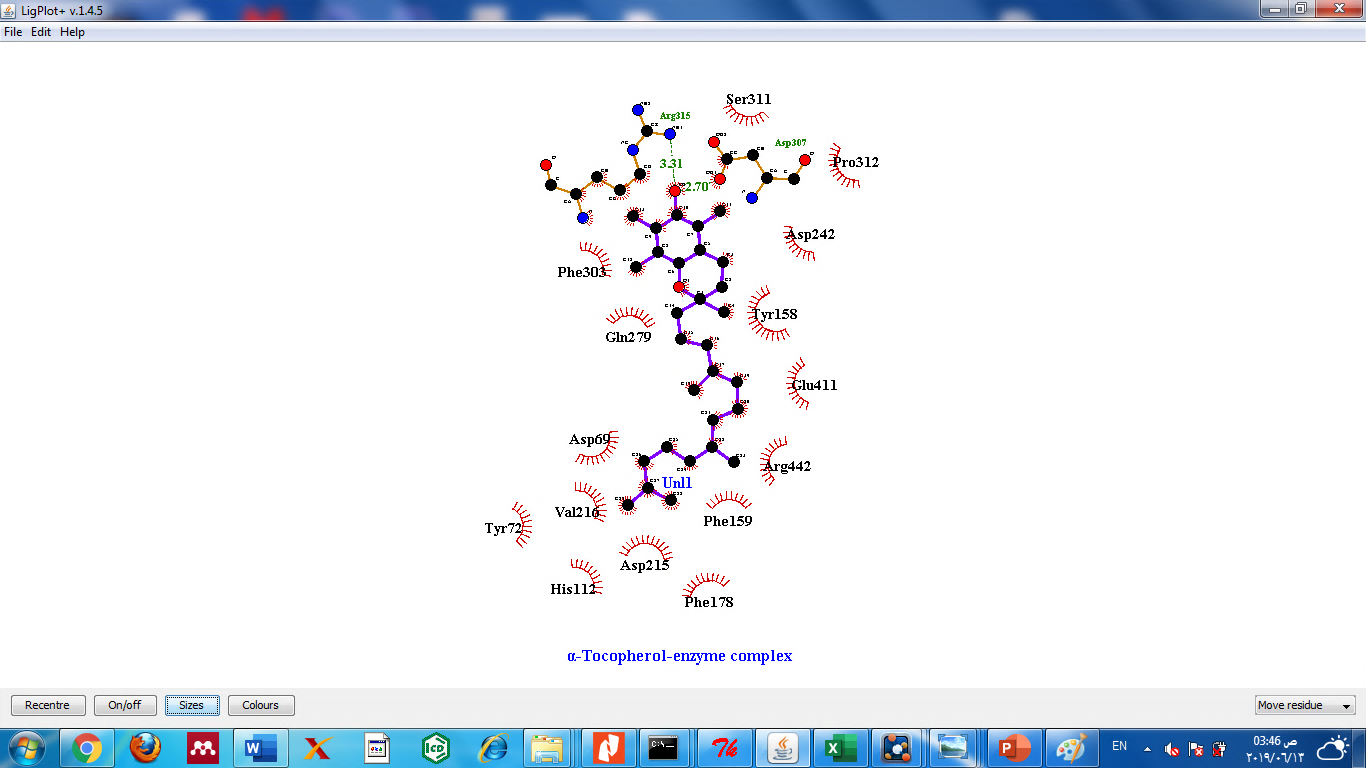


**α-Tocopherol**


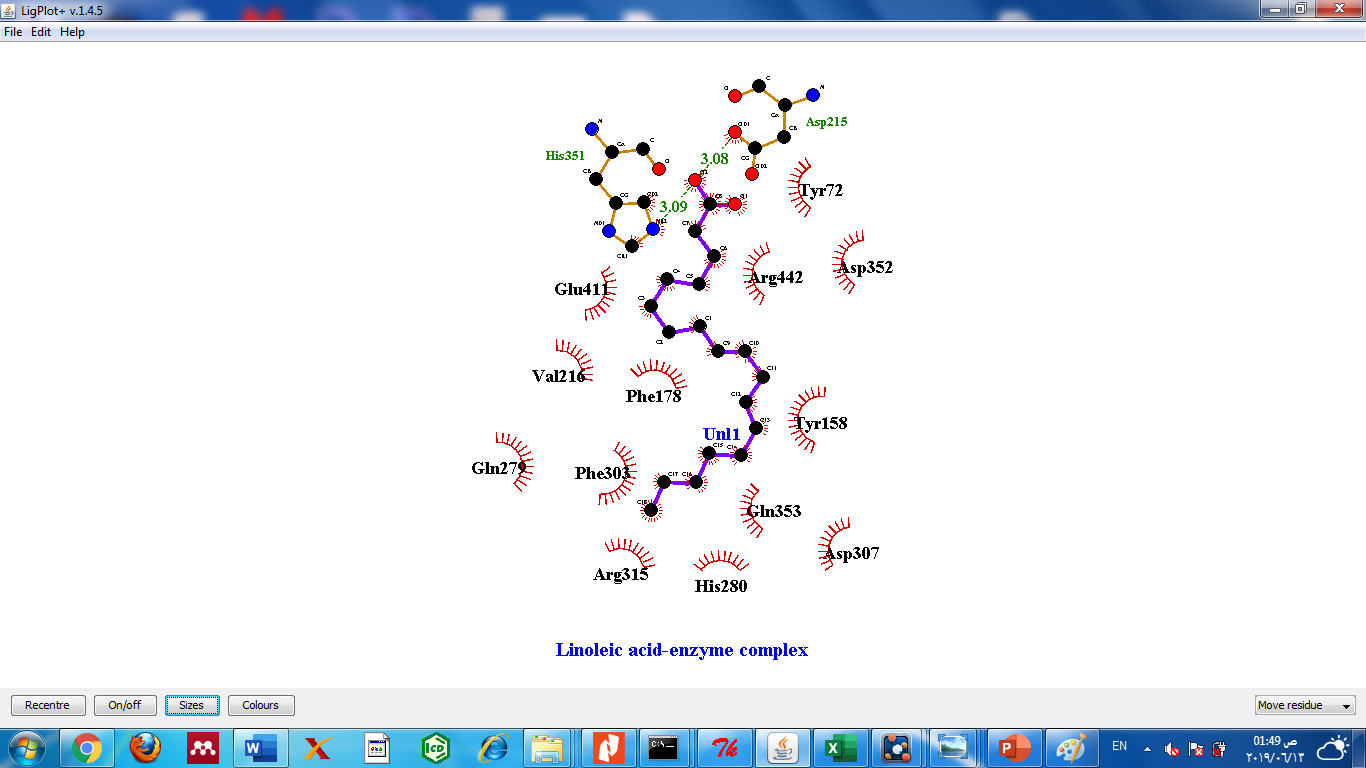


**Linoleic acid**


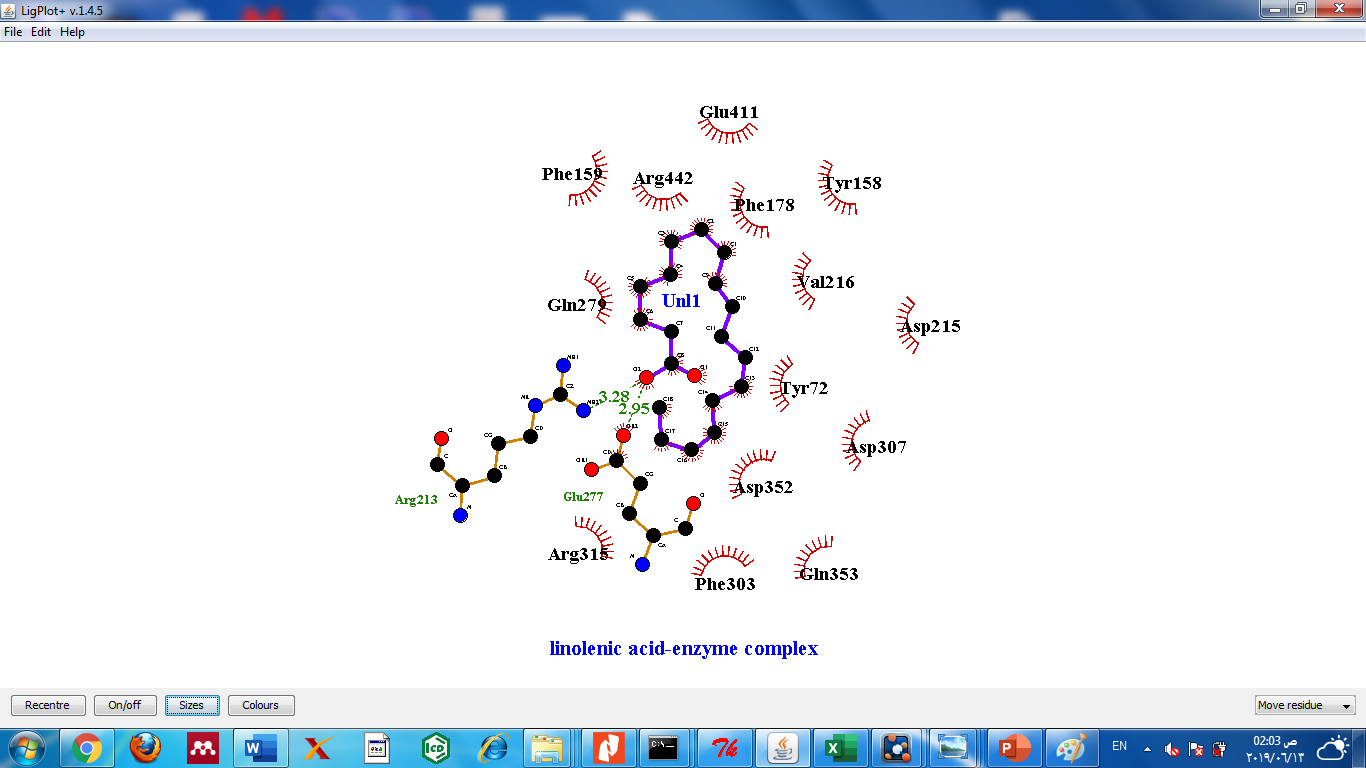


**α-Linolenic acid**


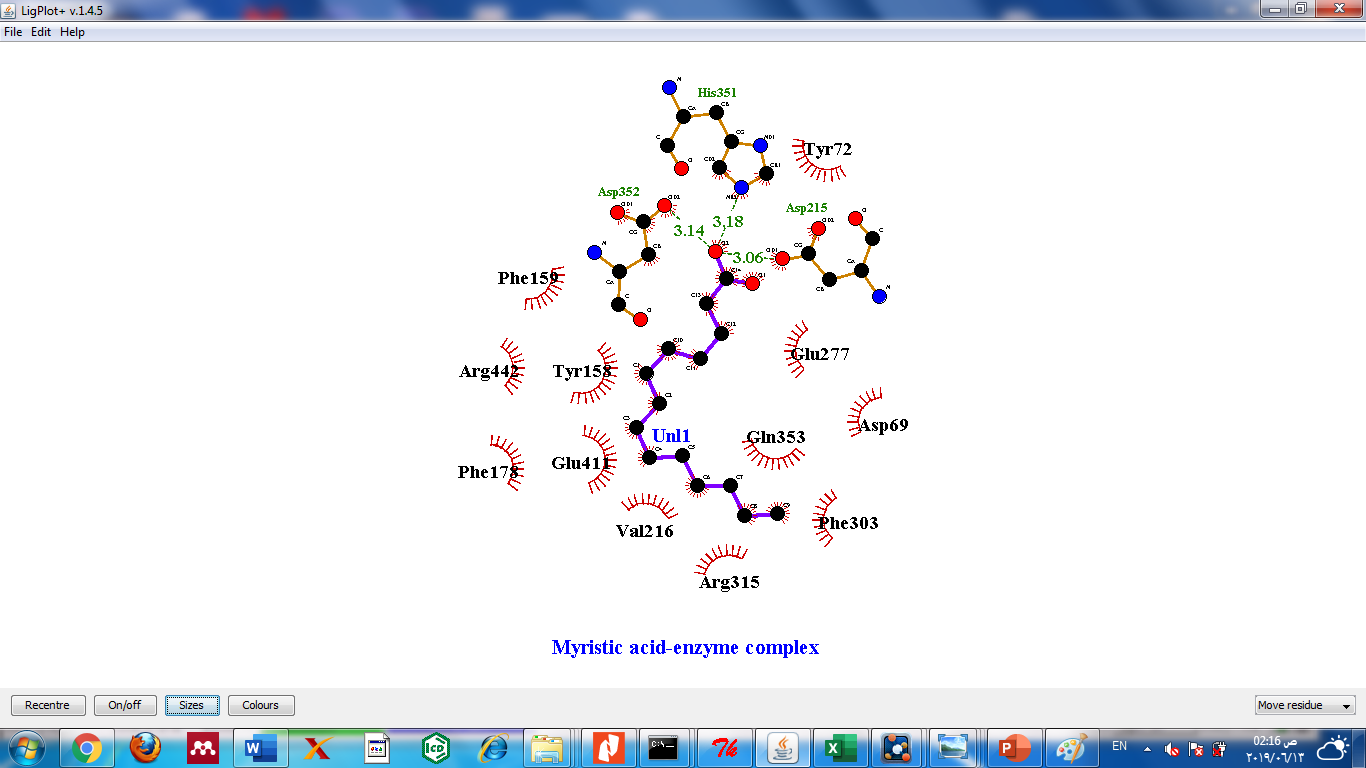


**Myristic acid**


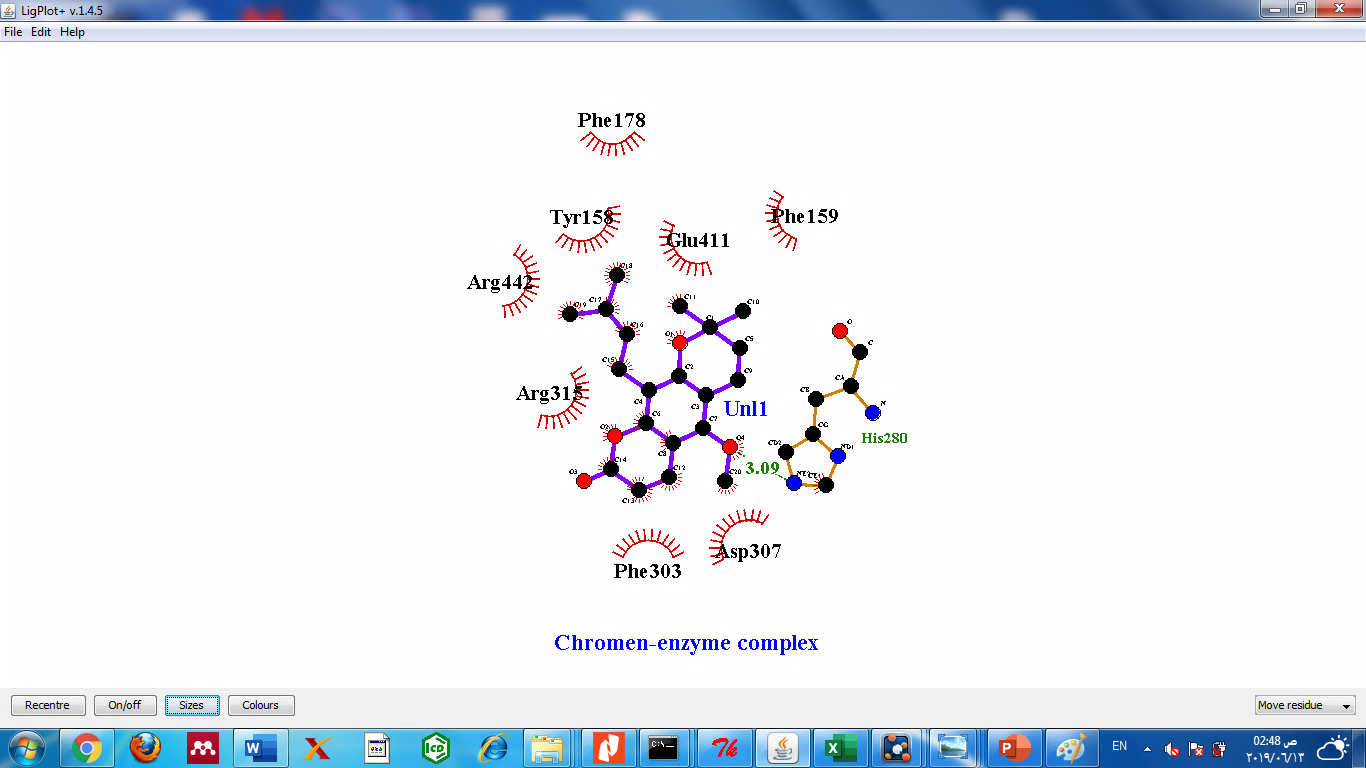


**5-Methoxy-8,8-dimethyl-10-(3-methyl-2-butenyl)-2H,8H-pyrano[3,2-g]chromen-2-one**
