# Supplementary figures and images for: Investigation of α-Glucosidase Inhibitory Metabolites from Tetracera scandens Leaves by GC–MS Metabolite Profiling and Docking Studies
Source: Biomolecules. 2020 Feb 12;10(2):287. doi: 10.3390/biom10020287 (PMC7072363; doi:10.3390/biom10020287)

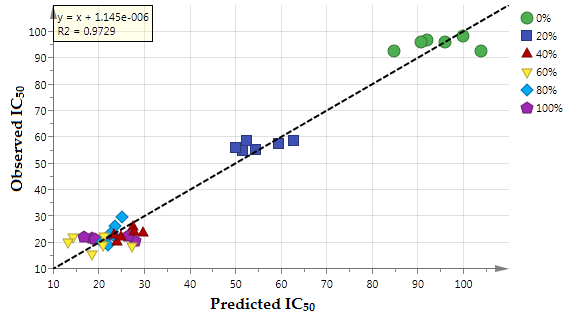

Supplement: Supplementary file 1 [file biomolecules-10-00287-s001.zip › Proofed Supplementary/Supplementary data (Figure S1).gif]
